# A rare case of cardiac paraganglioma presenting as anginal pain: a case report

**DOI:** 10.1186/1757-1626-2-72

**Published:** 2009-01-21

**Authors:** Tabindeh J Khalid, Omer Zuberi, Lara Zuberi, Imran Khalid

**Affiliations:** 1 Henry Ford Hospital, 2799 W Grand Blvd, Detroit, MI, 48202, USA

## Abstract

**Introduction:**

Primary cardiac paraganglioma is a very rare tumor with less than sixty reported cases in the literature. The clinical presentation is variable, but is most commonly manifested by hypertension and symptoms related to the catecholamine excess.

**Case Report:**

We report a case of a 35 year old man who presented with anginal pain and hypertension. He was found to have a cardiac mass on the computed tomographic scan and echocardiogram. He underwent surgical exploration of the mass which on biopsy was found to be a 'Cardiac Paraganglioma'. Surgical resection of the tumor was successfully done and the patient is doing well five years after the surgery without any evidence of recurrence. His blood pressure, however, failed to normalize and needed single agent antihypertensive therapy.

**Conclusion:**

Cardiac paragangliomas have a relatively favorable outcome if diagnosed and resected in time. We briefly review the literature regarding the diagnosis, treatment and prognosis of this rare tumor.

## Introduction

Paragangliomas, also known as extra-adrenal pheochromocytomas, are tumors derived from the neural crest cells. Primary cardiac paraganglioma is a very rare tumor with only less than sixty reported cases in the literature [1]. The clinical presentation is variable, but is most commonly manifested by hypertension and the symptoms related to the catecholamine excess, including palpitations, headache, and sweating [2,3]. Their most common location is the left atrium, with only four cases reported originating from the right atrium [3,4]. We report here a case of a primary cardiac paraganglioma where the only presenting symptom was anginal type chest pain. We also briefly review the literature regarding the diagnosis, treatment and prognosis of this rare tumor.

## Case presentation

A 35 year old obese African American male, a mechanic by profession and no significant previous medical problems, came to the emergency room with left sided chest pain that started the night before and slowly got worse. By the time he reached the emergency room, the pain had reached 8 out of a maximum of10 in intensity. The pain was precipitated and aggravated by a stressful situation at home. It radiated to his left shoulder and was not relieved by rest. Patient had a five pack-year history of smoking but quit 10 years ago. He would occasionally drink alcohol. On further questioning, the patient revealed that he had been having mild left sided chest pains, sharp in nature and similar to the current episode, for the last few months. The pain would occur either with stress or exertion. He did not seek medical attention before because the pain would be transient and go away on its own after a few minutes. He was not on any medications and did not use any pain relieving medicines either for his prior symptoms.

In the emergency room, patient was in mild distress and had a blood pressure of 165/81, something new for him. All the rest of the vital signs were normal and both the cardiac and pulmonary examination did not reveal any abnormality either. The basic laboratory work up was normal except a lipid profile showing elevated cholesterol of 238 mg/dl. The patient had an electrocardiogram that showed some premature supraventricular complexes without any ischemic changes. Cardiac biomarkers including serial troponin-I were also normal. The chest pain subsided in the emergency room with intravenous morphine. The patient was admitted to the hospital in the telemetry unit. He was found to have some runs of non-sustained ventricular tachycardia while in the hospital. He underwent a cardiac exercise stress test and an echocardiogram which revealed that patient had a normal ejection fraction but a dilated left atrium along with apical wall motion abnormality.

**Figure 1:**
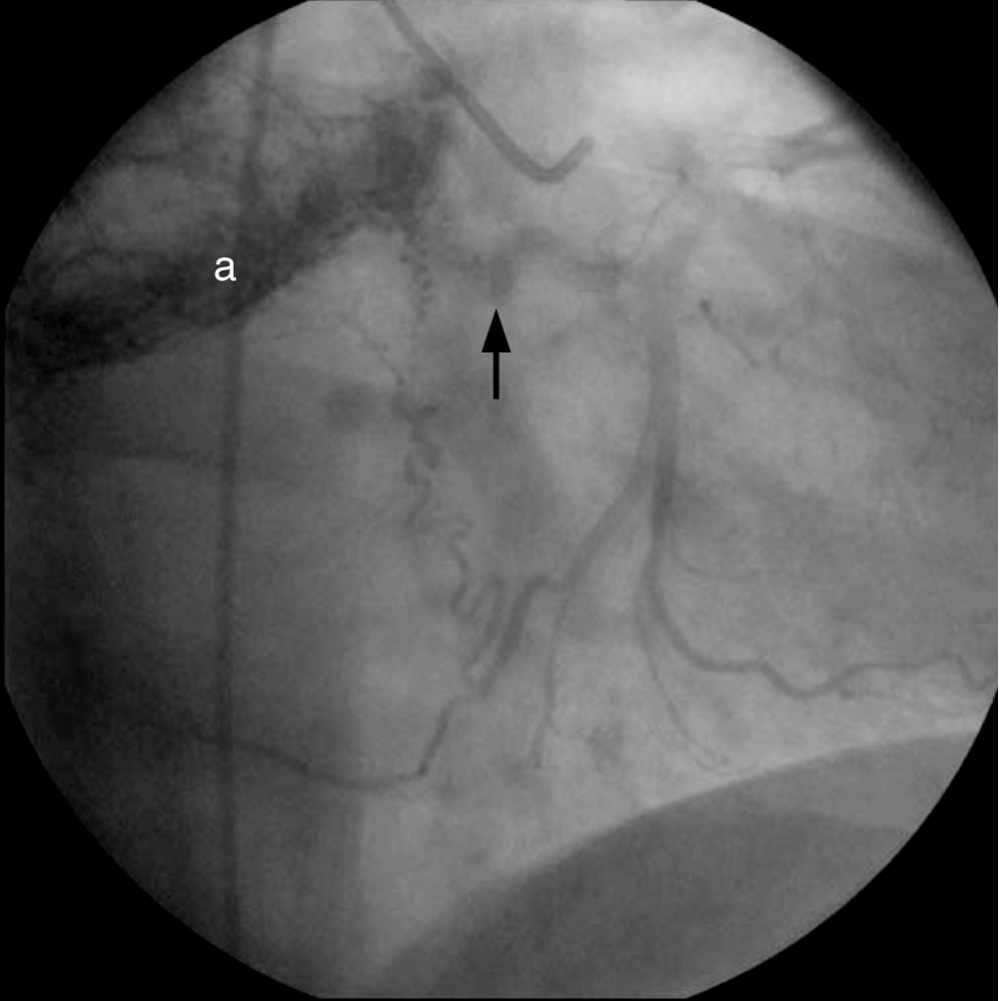
Cardiac catheterization image: showing the "tumor blush" (denoted by "a") with neovascularization seen projecting from the left circumflex artery (arrow) to the "tumor blush."

**Figure 2:**
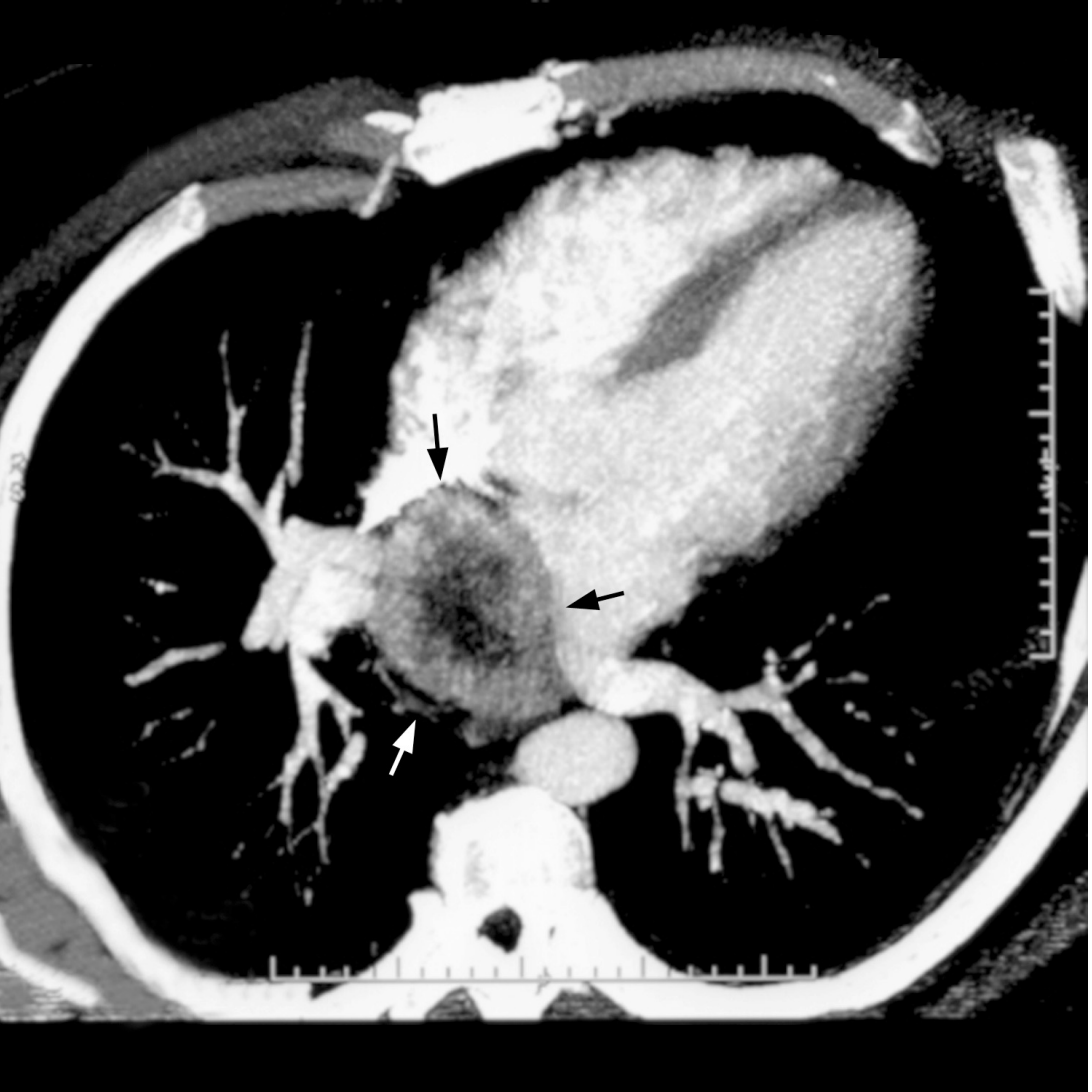
**CT scan: image showing a solid and likely necrotic mass (arrows) extending posterior to the left atrium**.

Patient then underwent a cardiac catheterization. It did not show any obstruction in the coronary arteries, but did reveal a "tumor blush" with neovascularization seen projecting from the left circumflex artery to the "tumor blush". (Figure 1) Following the cardiac catheterization, patient had a CT scan of the chest to evaluate for any mass or tumor. The CT scan showed a 6 cm solid and likely necrotic mass beginning slightly anterior to the carina and extending caudally posterior to the left atrium. (Figure 2) No prior imaging was available for comparison.

**Figure 3:**
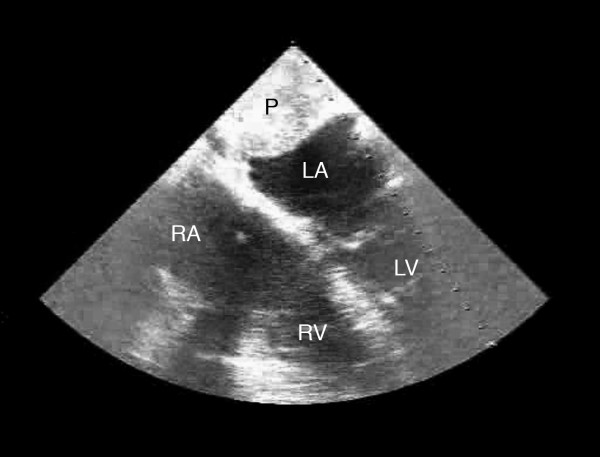
**Trans-esophageal echocardiogram: TEE shows a large mass, likely extrinsic to the heart, compressing the left atrium (LA)**. RA denotes Right Atrium; RV denotes Right Ventricle; LV denotes Left Ventricle; P denotes the Cardiac Paraganglioma.

**Figure 4:**
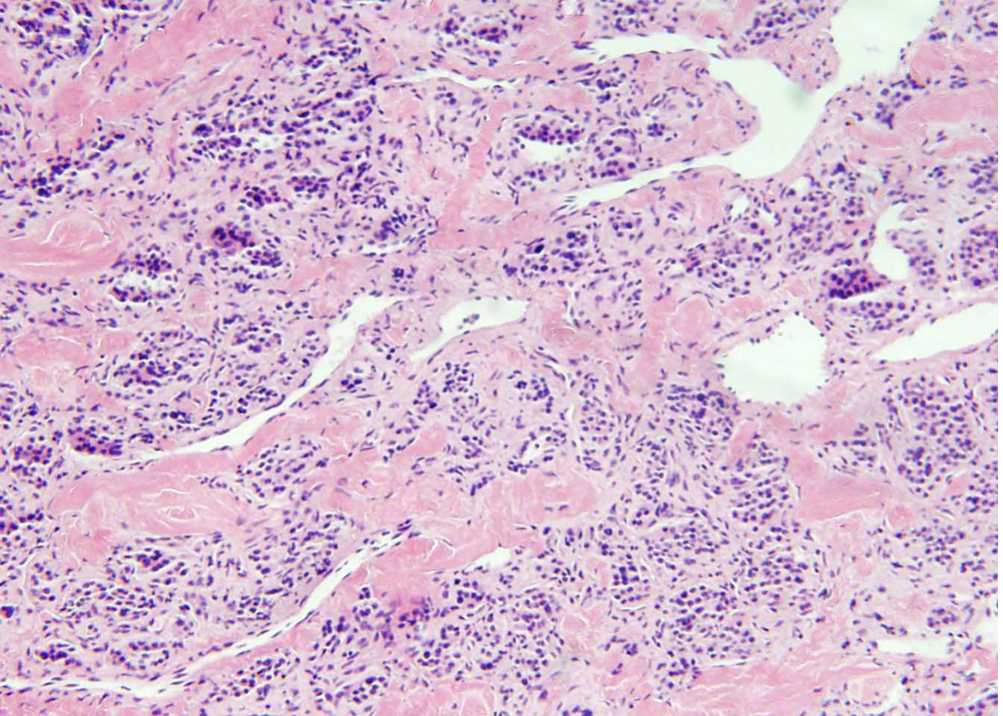
**Biopsy from the cardiac mass: photomicrograph of the biopsy specimen taken from the tumor showing tumor cells arranged in a nest-like (Zellballen) pattern, in a background of thick collagenous stroma, separated by thin vascular network**. (Original magnification ×40; H&E stain).

As it was still unclear whether the mass was originating intrinsic or extrinsic to the heart, so to define it better anatomically, a transesophageal echocardiogram (TEE) was performed. The TEE also showed the large mass, likely extrinsic to the heart, compressing the left atrium. (Figure 3) Finally, the patient underwent an open heart surgery for definitive diagnosis and management. During surgical exploration, an extremely hard mass was found on the dome of the left atrium, and on further exploration, the indurated tissue clearly extended to the left atrial appendage, underneath the superior vena cava and past the origin of the right superior pulmonary vein. Frozen sections of the biopsy from the tumor were suggestive of "Cardiac Paraganglioma", showing tumor cells arranged in a nest-like (Zellballen) pattern-separated by thin vascular network. (Figure 4) This was later confirmed further with special staining. The patient's tumor was initially deemed unresectable, because of its location and local invasion, by the operating surgeon and the median sternotomy incision was closed. However, patient then had a second opinion by a different surgeon who successfully resected the tumor and the patient recovered from the operation without any major complications. His normetanephrine levels which were very elevated before the tumor resection (1447 microgram/24 hours), normalized to 312 microgram/24 hours (normal value 110–620 microgram/24 hours) after the tumor was resected. Five years later he is doing well with no recurrence of the tumor, though he has to use a thiazide diuretic for blood pressure control.

## Discussion

Although most cardiac paragangliomas are benign, local invasion can occur and metastases have been reported in up to 10% of cases [5]. Localization of a cardiac paraganglioma represents a significant diagnostic challenge. Multiple techniques, including CT, MRI, iodine-123/131-meta-iodobenzylguanidine scintigraphy, indium-octreotide scintigraphy, coronary angiography, TEE, and staged venous sampling for catecholamines have been used to successfully localize the tumor [2]. The role of PET imaging in the diagnosis of paragangliomas is not yet well defined. When F-18 FDG PET has been studied in paragangliomas, tracer uptake has been variable. There are, however, reports of patients with F-18 FDG uptake within these tumors which did not initially take up I-123 MIBG [6]. In our patient, we used both CT and TEE to localize and define the tumor pre-operatively.

Taking in regard the anatomical location of the mass and elevated urinary normetanephrine levels, the patient was referred for surgical exploration. A recent report of a single center surgical experience has shown that the approach to remove the paragangliomas can be either a median sternotomy or posterolateral thoracotomy, depending on the location in the mediastium [7]. Our patient was deemed unsuitable for surgery on the initial attempt, however, he had a second opinion and the tumor was successfully resected utilizing median sternotomy and cardiopulmonary bypass to facilitate dissection of the paraganglioma from the heart.

The long-term outlook for patients with excised cardiac paragangliomas is unknown. In the only reported case series, there was one intraoperative death due to blood loss and ten of the 11 patients were alive at follow-up (median follow-up: 2.3 years; range, 0 to 19.4 years) [7]. Survival of up to 14 years, however, has been reported in a patient with excised mediastinal paraganglioma [8]. Our patient is doing well five years after his surgery.

After surgery, symptoms of catecholamine excess are usually eliminated or greatly improved in patients who had a functioning tumor preoperatively, although hypertension may or may not resolve [7,9]. Surveillance can be done by following serum normetanephrine levels, repeating CT scans and/or TEE, and most recently PET imaging [10]. The literature, however, offers no consensus on the treatment if the tumor does recur. Our patient did not have recurrence of the tumor five years after the excision as evidenced by a negative CT scan and normal normetanephrine levels. However, his hypertension that coincided with the diagnosis of the tumor five years ago has not resolved and he is on a thiazide diuretic with optimal blood pressure control.

## Abbreviations

TEE: Trans-esophageal echocardiography; CT: Computed Tomography; MRI: Magnetic Resonance Imaging; I-123 MIBG: Iodine-123-meta-iodobenzylguanidine scintigraphy.

## Consent

Written informed consent was obtained from the patient for publication of this case report and accompanying images. A copy of the written consent is available for review by the Editor-in-Chief of this journal.

## Competing interests

The authors declare that they have no competing interests.

## Authors' contributions

TJK and OZ wrote the case report part of the manuscript. LZ wrote the introduction and prepared the images. IK came up with the concept of the manuscript and wrote the discussion portion of the manuscript. All authors read and approved the final manuscript.
